# Less healthy, but more active: Opposing selection biases when recruiting older people to a physical activity study through primary care

**DOI:** 10.1186/1471-2458-8-182

**Published:** 2008-05-27

**Authors:** Tess J Harris, Christina R Victor, Iain M Carey, Rika Adams, Derek G Cook

**Affiliations:** 1 Division of Community Health Sciences, St George's, University of London, Cranmer Terrace, Tooting, London, SW17 ORE, UK; 2 Sonning Common Health Centre, Wood Lane, Sonning Common, Oxfordshire, RG4 9SW, UK; 3 School of Health and Social Care, Reading University, Whiteknights Lane, Reading, Berkshire, UK

## Abstract

**Background:**

Physical activity studies in older people experience poor recruitment. We wished to assess the influence of activity levels and health status on recruitment to a physical activity study in older people.

**Methods:**

Comparison of participants and non-participants to a physical activity study using accelerometers in patients aged ≥ 65 years registered with a UK primary care centre. Logistic regression was used to calculate odds ratios (OR) of participants in the accelerometer study with various adjustments. Analyses were initially adjusted for age, sex and household clustering; the health variables were then adjusted for physical activity levels and vice versa to look for independent effects.

**Results:**

43%(240/560) participated in the physical activity study. Age had no effect but males were more likely to participate than females OR 1.4(1.1–1.8). 46% (76/164) of non-participants sent the questionnaire returned it. The 240 participants reported greater physical activity than the 76 non-participants on all measures, eg faster walking OR 3.2(1.4–7.7), or 10.4(3.2–33.3) after adjustment for health variables. Participants reported more health problems; this effect became statistically significant after controlling for physical activity, eg disability OR 2.4(1.1–5.1).

**Conclusion:**

Physical activity studies on older primary care patients may experience both a strong bias towards participants being more active and a weaker bias towards participants having more health problems and therefore primary care contact. The latter bias could be advantageous for physical activity intervention studies, where those with health problems need targeting.

## Background

Physical activity studies on older people often recruit through primary care, such studies usually report low participation, both for surveys (46% [1], 57% [2]) and more markedly for intervention studies (26% [3], 32% [4], 35% [2]). Information about non-participants is lacking, but higher activity levels have been associated with increased recruitment [4,5], leading to potential selection bias and difficulties in generalizing results [6]. Unfortunately, these studies did not report on non-participants' health status. Whilst older people with higher activity levels tend to be healthier [7], recruiting through primary care could encourage those with more illnesses and primary care contact to respond, leading to bias in an opposing direction. Our objective was to compare the self-reported health status and physical activity levels of participants and non-participants in a primary care based physical activity study.

## Methods

As part of a randomized controlled trial of different recruitment strategies to a physical activity study, 560 patients ≥ 65 years registered with a primary health care centre (general practice) in Oxfordshire, UK were randomly selected by household [8]. Those living in care homes, those with dementia, terminal illness, poorly controlled cardiac failure or unstable angina and those housebound due to disability were first excluded by computer record search and by general (family) practitioner and district (community) nurse examination of registered patient lists. All 560 patients were invited to take part in a study measuring customary physical activity levels objectively for a 7-day period using motion sensors (accelerometers and pedometers). A random half (280) also received a 12-page questionnaire with their study information, asking details about physical health (general health, limiting longstanding illness [9], disability [10], pain [11], chronic disease [12], smoking status, weight and height), depressive symptoms [13], self-reported physical activity levels [14,15] and attitudes towards physical activity [16] (See Additional file 1). Subjects were encouraged to return the questionnaire, whether or not they participated in the physical activity study, thus allowing a comparison of participants and non-participants. Those participating who had not been randomized to receive a questionnaire, completed one at their baseline assessment.

### Ethical approval & informed consent

Ethics committee approval for the study was given by Oxfordshire REC A (reference no. 06/Q1604/94). The patient information sheets sent to all 560 individuals explained the study in detail, including the use of questionnaire information provided by those returning questionnaires, but not wanting to participate further. Full informed written consent was obtained from the 240 participating in the physical activity study.

### Analysis

Logistic regression was used to estimate odds ratios for participating in the accelerometer study, adjusting for age and sex as appropriate and household clustering, using the cluster option in STATA 9 [16]. For age and sex comparisons the analyses were based on all 520 subjects. For analyses examining health and physical activity from the brief questionnaire, the comparison was based on all participants to the accelerometer study and those non-participants who received a questionnaire and returned it. Analyses were adjusted for age, sex and household clustering. Health measures were adjusted additionally for the effect of physical activity (walking pace and number of hours walked in the last week) and physical activity measures for the effect of health (limiting long-standing illness, number of chronic diseases, disability, falls and chronic pain) in order to see whether any effects were independent. To check that it was reasonable to combine postal questionnaires on participants with those completed at baseline assessment, a comparison of participants and non-participants as above was repeated, restricted to those randomised to postal questionnaires.

## Results

Recruitment rate to the physical activity study was 43%(240/560). Comparison overall of participants (n = 240) and non-participants (n = 320), adjusted for age, sex and household clustering, showed that males were more likely to participate OR 1.4(95% C.I. 1.1–1.8). There was no statistically significant association between age and participating, baseline age 65–69 OR = 1, age 70–79 OR 0.8(95% C.I. 0.5–1.1), age = 80 OR 0.8(95% C.I. 0.5–1.4).

**Figure 1:**
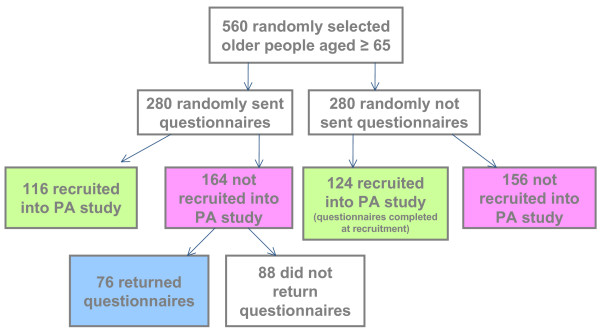
Participant flow through the study.

Of the 280 people sent a postal questionnaire, 116 were recruited to the study and 164 were not, of these non-participants 76/164(46%) completed the questionnaire (see Figure 1). Table 1 shows the comparison of participants (n = 240) (116 from the postal questionnaire group, 124 who completed the questionnaire at baseline assessment) and non-participants (n = 76) who returned the questionnaire. The results for age and sex are very similar to those for participants and non-participants overall.

**Table 1:** Comparison of non-participants & participants of physical activity study amongst questionnaire responders

	**Non-participants N = 76 n (%)**	**Participants N = 240 n (%)**	**Crude OR for participating (95% CI)**	**OR (95% CI) adj for age, sex, household clustering**^**1**^	
**DEMOGRAPHIC**					
**Sex**					
Female	45 (59.2)	115 (47.9)	1	1	
Male	31 (40.8)	125 (52.1)	1.6 (0.9–2.7)	1.6 (1.1–2.4)	
**Age**					
65–69	23 (30.3)	87 (36.3)	1	1	
70–79	40 (52.6)	111 (46.3)	0.7 (0.4–1.3)	0.8 (0.4–1.4)	
80 or more	13 (17.1)	42 (17.5)	0.9 (0.4–1.9)	0.8 (0.3–2.0)	

**SELF-REPORTED HEALTH**					**OR (95% CI) adj for age, sex, household clustering & self-reported activity**^**2**^

**General Health**					
Very good/Good	58 (84.1)	200 (85.1)	1	1	1
Fair/bad	11 (15.9)	35 (14.9)	0.9 (0.7–1.4)	1.1 (0.7–1.6)	1.3 (0.9–2.0)
**Limiting longstanding illness**					
No	53(77.9)	176 (73.6)	1	1	1
Yes	15 (22.1)	65 (28.6)	1.4 (0.7–2.7)	1.6 (0.8–3.3)	2.9 (1.2–7.1)
**Disability**					
No	40 (53.3)	125 (52.7)	1	1	1
Yes	35 (46.7	112 (47.3)	1.0 (0.6–1.7)	1.3 (0.7–2.5)	2.4 (1.1–5.1)
**Chronic pain**					
No	46 (66.7)	146 (63.5)	1	1	1
Yes	23 (33.3)	84 (36.5)	1.2 (0.7–2.0)	1.4 (0.7–2.6)	2.1 (1.0–4.5)
**Chronic disease**					
No	25 (32.9)	55 (22.9)	1	1	1
Yes	51 (67.1)	185 (77.1)	1.6 (0.9–2.9)	1.7 (0.9–3.0)	1.8 (1.0–3.2)
**Use a walking aid**					
No	66 (89.2)	217 (91.9)	1	1	1
Yes	8 (10.8)	19 (8.1)	0.7 (0.3–1.7)	0.8 (0.3–2.1)	1.4 (0.5–3.8)
**Fallen in last year**					
No	56 (76.7)	169 (71.9)	1	1	1
Yes	17 (23.3)	66 (28.1)	1.3 (0.7–2.4)	1.6 (0.8–2.9)	2.0 (1.0–4.0)
**Current smoker**					
No	72 (96.0)	221 (94.0)	1	1	1
Yes	3 (4.0)	14 (6.0)	1.5 (0.4–5.4)	1.3 (0.3–5.1)	1.5 (0.4–5.1)
**Body Mass Index**					
Normal weight	28 (46.7)	97 (43.1)	1	1	1
Overweight or obese	32 (53.3)	128 (56.9)	1.2 (0.7–2.0)	1.2 (0.7–2.4)	1.4 (0.7–2.6)
**Geriatric Depression Score**^**3**^					
<4	67 (88.2))	213 (89.5)	1	1	1
4 or more	9 (11.8)	25 (10.5)	0.9 (0.4–2.0)	0.9 (0.4–2.0)	1.1 (0.5–2.5)

**SELF-REPORTED ACTIVITY**					**OR (95% CI) adj for age, sex, household clustering & self-reported health**^**4**^

**Walking pace compared to others**					
Slower/much slower	16 (21.3)	22 (9.4)	1	1	1
About the same	34 (45.3)	100 (42.5)	2.1 (1.1–4.5)	1.9 (1.0–4.2)	4.6 (1.7–12.5)
Faster/much faster	25 (33.3)	113 (48.1)	3.3 (1.5–7.1)	3.2 (1.4–7.7)	10.4 (3.2–33.3)
**Hours walked in last week?**					
None	24 (31.6)	37 (15.4)	1	1	1
Up to 2 hours	25 (32.9)	87 (36.3)	2.3 (1.1–4.5)	2.3 (1.1–4.7)	2.5 (1.1–5.6)
More than 2 hours	27 (35.5)	116 (48.3)	2.8 (1.4–5.4)	2.7 (1.3–5.5)	2.8 (1.3–6.1)
**Average hours gardening weekly**					
None	19 (25.3)	23 (10.0)	1	1	1
Up to 3.5 hrs/week	30 (40.0)	102 (44.4)	2.8 (1.4–5.8)	2.7 (1.3–5.6)	2.7 (1.1–6.9)
>3.5 hrs/week	26 (34.7)	105 (45.7)	3.3 (1.6–7.0)	2.8 (1.3–6.3)	3.3 (1.2–8.7)
**Activity compared to others**					
Less active or the same	27 (35.5)	49 (20.6)	1	1	1
More active	33 (43.4)	116 (48.7)	1.9 (1.1–3.6)	1.9 (1.0–3.6)	2.2 (1.0–4.5)
Far more active	16 (21.1)	73 (30.7)	2.5 (1.2–5.1)	2.3 (1.1–5.2)	3.1 (1.3–7.0)
**Do you cycle?**					
No	63 (87.5)	187 (78.9)	1	1	1
Yes	9 (12.5)	50 (21.1)	1.9 (0.9–4.0)	1.7 (0.7–4.1)	2.0 (0.8–5.1)
**Do you walk a dog?**					
No	67 (88.2)	184 (78.6)	1	1	1
Yes	9 (11.8)	50 (21.4)	2.0 (0.9–4.3)	2.0 (0.8–5.2)	2.0 (0.7–5.2)
**Do you do heavy housework?**					
No	23 (34.3)	50 (22.7)	1	1	1
Yes	44 (65.7)	170 (77.3)	1.8 (1.0–3.2)	1.7 (1.0–3.2)	2.3 (1.2–4.5)
**Positive attitudes towards activity?**					
Low	35 (53.9)	73 (31.6)	1	1	1
High	30 (46.2)	158 (68.4)	2.5 (1.4–4.4)	2.5 (1.4–4.5)	3.4 (1.8–6.6)

^1^Household clustering – adjusted for the clustering by 237 households.

^2^Self-reported physical activity- adjusted for effect of walking pace and hours walked in last week

^3^Geriatric Depression Score 15 items, using cut-off <4/≥4, which gives a sensitivity of 91% and specificity of 72% for detecting major depression [13].

^4^Self-reported health – adjusted for effect of limiting longstanding illness, chronic disease, disability, falls & pain.

After adjusting for age, sex and household clustering, participants tended to report more health problems than non-participants for most variables, (but no differences were statistically significant at p = 0.05). The exceptions were: use of a walking aid which showed a non-significant effect in the opposite direction; and depression score which was unrelated to participation. Adjusting for self-reported physical activity levels strengthened the associations between poorer health and participating such that those reporting limiting longstanding illness, disability, chronic pain, chronic disease and a fall in the last year were more likely to participate.

After adjusting for age, sex and household clustering, participants reported more physical activity than non-participants for all measures and more positive attitudes towards physical activity. Apart from cycling and dog-walking, where numbers were small, these differences were all statistically significant and several showed dose-response effects. Adjusting these estimates for self-reported health strengthened these effects still further.

Analyses restricted to participants (116) and non-participants (76) who returned the postal questionnaire showed very similar findings in terms of effect estimates to those presented, based on all questionnaire completers. Although the confidence intervals were wider due to smaller numbers, several associations with participation reached statistical significance at p = 0.05 for both health, e.g. disability, adjusted OR 2.4 (05% C.I. 1.1–5.5) and physical activity variables, e.g. higher activity levels than others, adjusted OR 3.1 (95% C.I. 1.1–8.7).

## Discussion

Recruitment to our motion sensor activity study was 43%, higher than for physical activity intervention studies in this age group [2-4], but lower than for surveys [1,2]. With this recruitment level, estimation of potential non-response bias is important.

Our findings suggest two separate issues leading to potential bias. Firstly, participants were more physically active than non-participants (consistent with men being more likely to participate). This has been reported in other physical activity studies in older people [3-5]. Secondly, participants reported more health problems than non-participants. This effect opposes the physical activity effect, and was much clearer after controlling for self-reported physical activity. At first, these findings seem counter-intuitive. Other studies in older people have reported that participants have better health than non-participants [18-20], suggesting a "healthy volunteer" effect, although Ives et al found participants were more likely to have a disease history and use health services more [21]. However, it seems plausible that recruiting older people to studies through primary care may lead to those with increased contact with primary care (ie more illnesses) to be more likely to take part. This fits with other work showing that older primary care patients with poorer physical and psychological health and greater primary care service use were more likely to give consent for their health records to be accessed for research [22]. Unfortunately, the other primary care based studies showing differences in physical activity levels between participants and non-participants did not report on health or functional ability [3-5].

An important weakness of our study was that self-reported health and physical activity details were only available on those non-participants who completed the questionnaire. The similarities seen in age and sex comparisons between participants and non-participants overall and in those responding to the questionnaire is reassuring and suggests that the non-participant questionnaire responders are representative of non-participants, at least in terms of age and sex, although they could still differ in other important ways (such as activity level or health) that we lack information on. The similarities found when restricting analyses to only those sent postal questionnaires, confirms that it was reasonable to include participants in the analysis who completed the questionnaire at their baseline assessment.

## Conclusion

Physical activity studies on older people recruited from primary care settings may be biased by two opposing issues which need consideration when generalizing the results: a strong bias towards participants being more physically active, and a weaker bias towards participants having more health problems and therefore likely primary care contact. This latter bias could be used to advantage when considering interventions to increase physical activity, where those who are least active and those with more physical health problems need targeting most.

## Competing interests

The authors declare that they have no competing interests.

## Authors' contributions

TJH, CRV and DGC designed the study. TJH and RA collected data. TJH, IMC and DGC were involved in analysis and interpretation of data. All authors were involved in drafting the manuscript and revising it critically for important intellectual content. All authors have given final approval of the version to be published.

## Pre-publication history

The pre-publication history for this paper can be accessed here:



## Supplementary Material

Additional file 1 supplementary questionnaire harris2008. Questionnaire on health and self-report physical activity levels. Click here for file
