# Supplementary material for: Less healthy, but more active: Opposing selection biases when recruiting older people to a physical activity study through primary care
Source: BMC Public Health. 2008 May 27;8:182. doi: 10.1186/1471-2458-8-182 (PMC2426698; doi:10.1186/1471-2458-8-182)
Supplement: Additional file 1 — supplementary questionnaire harris2008. Questionnaire on health and self-report physical activity levels. [file 1471-2458-8-182-S1.doc]

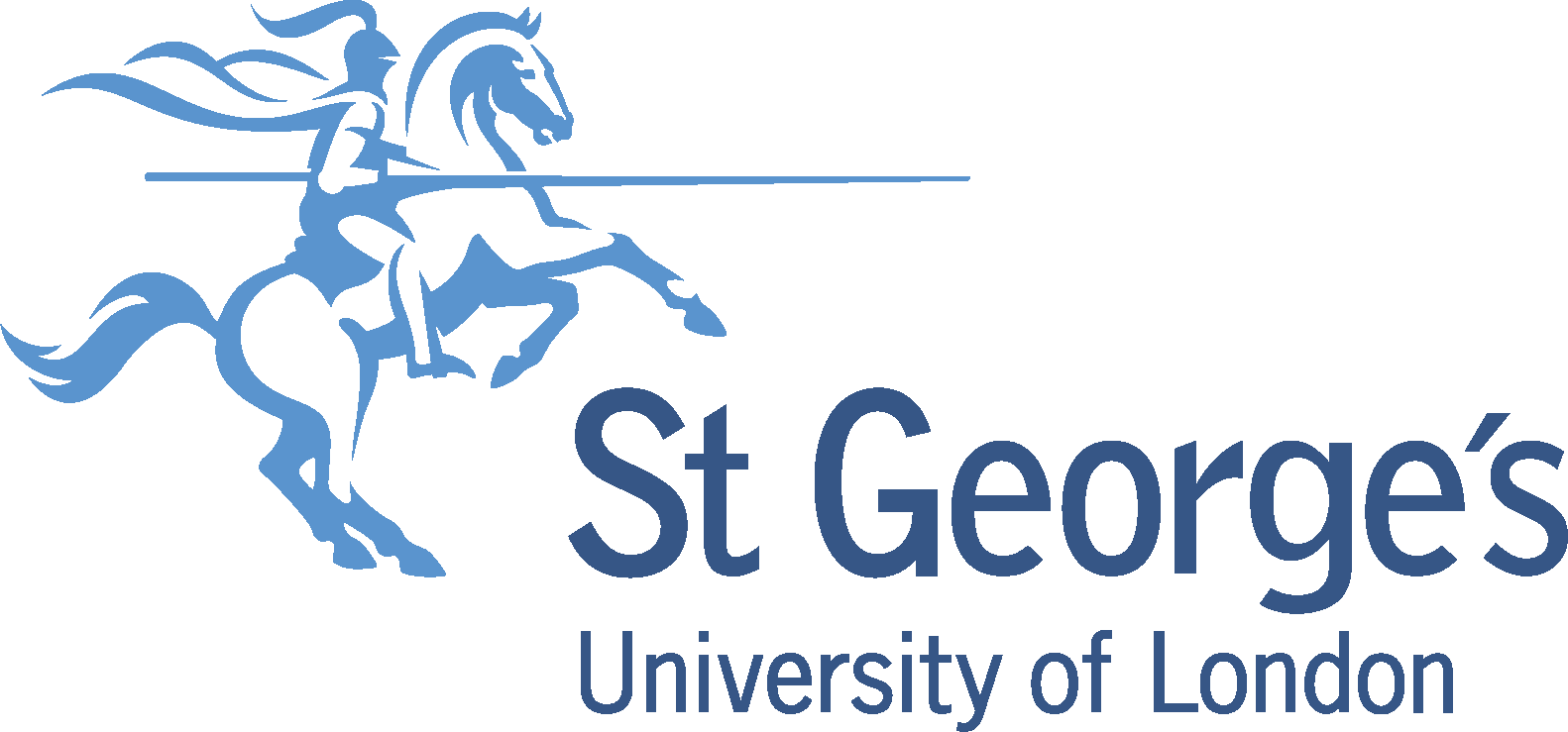

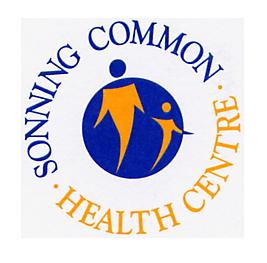


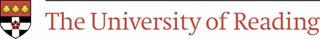


***PACE*** *(****P****hysical* ***A****ctivity in* ***C****ommunity* ***E****lderly)*

***Health and physical activity survey***

__________________________________________________________

**Thank you for filling in this questionnaire. It will probably**

**take you about 10 minutes to complete.**

**Please answer each question as best you can.**

**Please feel free to write comments by any question.**

**All information will be treated in the strictest confidence.**

**Please return the completed questionnaire in the freepost envelope provided.**

**Please enter your date of birth ____ / ____ / _____**

## Thank you

Study IDNO __________

**Please put a tick in the box next to the most appropriate answer for each question.**

✓

## Some general questions about your health

### How is your health in general?

very good ⬜

good ⬜

fair ⬜

bad ⬜

very bad ⬜

### Do you have any long-standing illness, disability or infirmity?

### By long-standing, we mean anything that has troubled you over a period of time, or that is likely to affect you over a period of time?

Yes ⬜ If Yes, go to question 3

No ⬜ If No, go to question 4

### Do any of these illnesses or disabilities limit your activities in

### any way?

###

### Yes ⬜

### No ⬜

**4 Approximately how tall are you?** ………………………………………

**5 Approximately how much do you weigh?** ......................................

## Specific questions about your health

**Have you *ever* been told by a doctor or nurse that you have any of these conditions? (Please tick all that apply to you)**

**Yes**

### Angina ………………………………..…….. ⬜

### A heart attack ………………….…..….….. ⬜

### Other heart problems………..…..…..…….. ⬜

### Stroke…………………………….…..……… ⬜

### High blood pressure…………….….………..⬜

### Chronic bronchitis…………………..…..…… ⬜

### Asthma ……………………….…..……..…… ⬜

### Diabetes……………………………..…..…… ⬜

### Arthritis ………….…..…..…………………….⬜

### Cancer (apart from skin cancer) ..……….. ⬜

### Depression…….…..……………………….. .⬜

### Parkinson’s Disease….……………..……… ⬜

### 13 How much physical or bodily pain have you had in the past 4 weeks?

None ⬜

Very mild or mild ⬜

Moderate ⬜

Severe or very severe ⬜

### 14 In the past four weeks, how much did pain interfere with your normal activities?

Not at all ⬜

A little bit ⬜

Moderately ⬜

Quite a bit or extremely ⬜

### 16 Can you see well enough to recognise a friend across a road ?

Yes, without glasses ⬜

Yes, with glasses ⬜

No ⬜

### 17 In the last year have you fallen over?

Yes ⬜ Please go to question 18

No ⬜ Please go to question 19

# 18 How many times have you fallen over in the last year?

Once ⬜

Less than 12 times ⬜

12 times or more ⬜

Not sure ⬜

**19 Do you regularly use a walking stick, walking frame or**

**wheelchair to move around?**

Yes ⬜ No ⬜

**20 Do you take four or more different medications every day?**

Yes ⬜ No ⬜

**21 Have you ever smoked?**

Yes ⬜ No ⬜

**22 Do you currently smoke?**

Yes ⬜

No ⬜

## Section C - Some questions about difficulties you may have

**Here are a few things people find difficult to do without help.**

**Do you or *would* you have difficulty with these activities?**

|  |  | No | Some | Unable |
| --- | --- | --- | --- | --- |
|  |  | Difficulty | Difficulty | to do alone |

### 1 Washing yourself all over ⬜ ⬜ ⬜

### 2 Cutting your own toenails ⬜ ⬜ ⬜

### 3 Getting on a bus ⬜ ⬜ ⬜

### 4 Going up and down stairs ⬜ ⬜ ⬜

### 5 Doing heavy housework ⬜ ⬜ ⬜

### 6 Shopping & carrying heavy bags ⬜ ⬜ ⬜

### 7 Preparing and cooking a hot meal ⬜ ⬜ ⬜

### 8 Reaching an overhead shelf ⬜ ⬜ ⬜

### 9 Tying a good knot in a piece of string ⬜ ⬜ ⬜

**10 Do you live alone?** Yes ⬜

No ⬜

## Section D - Some questions on how you feel

**The next questions ask about your feelings and mood.**

***Choose the answer for how you felt over the last week*.**

**Please answer all the questions**.

Yes No

### Are you basically satisfied with your life?……………………. ⬜ ⬜

### Have you dropped many activities and interests?…………… ⬜ ⬜

### Do you feel your life is empty?………………………………… ⬜ ⬜

### Do you often get bored?……………………………………… ⬜ ⬜

### Are you in good spirits most of the time?…………………… ⬜ ⬜

### Are you afraid that something bad is going to happen to you?⬜ ⬜

### Do you feel happy most of the time?……………………………⬜ ⬜

### Do you often feel helpless?………………………………………⬜ ⬜

### Do you prefer to stay home

rather than going out and doing new things?………………… .⬜ ⬜

### 10. Do you feel that you have more problems

with your memory than most? ………………………………. ⬜ ⬜

### Do you think it is wonderful to be alive now?………………. ⬜ ⬜

### Do you feel pretty worthless the way you are now?……… ⬜ ⬜

### Do you feel full of energy?…………………………………. ⬜ ⬜

### Do you feel that your situation is hopeless?………………. ⬜ ⬜

### Do you think that most people are better off than you are?…⬜ ⬜

16 Do you feel lonely?

All the time ⬜

Often ⬜

Sometimes ⬜

Never ⬜

**Section E - Some questions about physical activity**

**The questions asked in this part concern your daily activities**

**1 Are you able to walk outside the home?**

Yes ⬜

No, because I use a wheelchair ⬜

No, for a different reason, namely…………………

………………………………………………………..

If you are unable to walk / cycle, you can go on to question 8

**2 How many times did you take a walk outside during the last week?** ………………………………………….….times last week

**3 How long did such a walk usually last?** …………..minutes

**4 How would you describe your walking pace?**

Slow ⬜

Average ⬜

Brisk ⬜

**5 Did you take a walk that lasted longer than 1 hour during the last month?**

No ⬜

Yes ⬜

**5a If yes, how many times did you do that?** ……….times last month

**6 Do you walk a dog?**

No ⬜

Yes ⬜

**7 Do you ride a bicycle?**

No ⬜ (please go to question 8)

Yes ⬜

**7a If yes, how many times did you cycle last week?** …….…times

**7b How long on average did you cycle for each time?** ..…minutes

**7c How would you describe your cycling pace?**

Slow ⬜

Average ⬜

Fast ⬜

**8 Do you have a garden?**

No ⬜ (please go to question 9)

Yes ⬜

**8a If yes, how many hours, on average, a week do you spend doing gardening?**

In summer…………………hours

In winter……………………hours

**9 Do you do odd jobs in and around the house yourself (**e.g. painting and carpentry)?

No ⬜

Yes ⬜

**9a If yes, for how many hours a week?** …..…………hours weekly

**10 Do you do light housework, such as dusting and washing dishes?**

No ⬜

Yes ⬜

**10a If yes, for how many hours a week?** …..…………hours weekly

**11 Do you do heavy housework, such as vacuuming, scrubbing floors, cleaning windows?**

No ⬜

Yes ⬜

**11a If yes, for how many hours a week?** …..………..hours weekly

**12 Have you participated in any sporting activities recently?**

No ⬜

Yes ⬜

**12a If yes, what kind of sporting activity?** ……………………..

**12b How many hours on average, do you spend participating in sporting activities monthly?**

Less than 1 hour per month ⬜

……………… hours per month ⬜

**13 Do you have a hobby (other than gardening or sports)?**

No ⬜

Yes ⬜

**13a If yes, what kind of hobby?**

…………………………………………………………………………..

**13b How many hours a week do you spend on it?**

Less than 1 hour per week ⬜

……………… hours per week ⬜

**14 How often did you perspire during physical activity in the last week?**

Never ⬜

1-2 times ⬜

3-4 times ⬜

5 or more times ⬜

**15 Do you have a staircase in your home?**

No ⬜

Yes ⬜

**15a Do you climb stairs regularly (at least once per day)?**

Yes ⬜

No ⬜

**16 Most people of your age spend about 1 hour per day doing domestic work, odd jobs, gardening, walking and other physical activities. How do you compare with them?**

Far more active ⬜

More active ⬜

About the same ⬜

Less active ⬜

Far less active ⬜

**17 What do you think of your walking pace compared with other people of your age?**

Much faster ⬜

Faster ⬜

About the same ⬜

Slower ⬜

A lot slower ⬜

**18 How many hours, on average, do you sleep at night**

……………………………………………………………..…..hours

**19 How many hours do you sleep during the day** (eg, take a nap)?

I do not sleep in the daytime ⬜

I sleep for ……………….hours ⬜

**20 How many hours per day on average, do you watch television for?**

None ⬜

Less than 1 hour per day ⬜

……………… hours per day ⬜

**21 How many hours per day on average, do you use a computer for?**

None ⬜

Less than 1 hour per day ⬜

……………… hours per day ⬜

**Section F - Some questions about your attitudes to physical activity and health**

**Please indicate how strongly you agree or disagree with each statement**

**Strongly Slightly Unsure Slightly Strongly**

**Agree Agree Disagree Disagree**

1. Doing physical ⬜ ⬜ ⬜ ⬜ ⬜

activity is

satisfying and

rewarding to me

2 Doing physical ⬜ ⬜ ⬜ ⬜ ⬜

activity regularly

is good for me

3 There is very little ⬜ ⬜ ⬜ ⬜ ⬜

I can do to make

up for the physical

losses that come

with age

4 Keeping physically ⬜ ⬜ ⬜ ⬜ ⬜

active can

be helpful to my

health

**This page is for any other comments you may have on your health or this questionnaire**

**Thank you for filling in this questionnaire.**
